# Is Our Self Related to Personality? A Neuropsychodynamic Model

**DOI:** 10.3389/fnhum.2018.00346

**Published:** 2018-10-04

**Authors:** Andrea Scalabrini, Clara Mucci, Georg Northoff

**Affiliations:** ^1^Department of Psychological, Health and Territorial Sciences (DiSPuTer), G. d’Annunzio University of Chieti-Pescara, Chieti, Italy; ^2^University of Ottawa Institute of Mental Health Research and University of Ottawa Brain and Mind Research Institute, Ottawa, ON, Canada; ^3^Mental Health Centre, Zhejiang University School of Medicine, Hangzhou, China; ^4^Centre for Cognition and Brain Disorders, Hangzhou Normal University, Hangzhou, China; ^5^TMU Research Centre for Brain and Consciousness, Shuang Ho Hospital, Taipei Medical University, Taipei, Taiwan; ^6^Graduate Institute of Humanities in Medicine, Taipei Medical University, Taipei, Taiwan

**Keywords:** neuroscience, psychoanalysis, self-relatedness, resting state fMRI, personality organization, rest-self overlap/containment

## Abstract

The concept and the assessment of personality have been extensively discussed in psychoanalysis and in clinical psychology over the years. Nowadays there is large consensus in considering the constructs of the self and relatedness as central criterions to assess the personality and its disturbances. However, the relation between the psychological organization of personality, the construct of the self, and its neuronal correlates remain unclear. Based on the recent empirical data on the neural correlates of the self (and others), on the importance of early relational and attachment experiences, and on the relation with the brain’s spontaneous/resting state activity (rest–self overlap/containment), we propose here a multilayered model of the self with: (i) relational alignment; (ii) self-constitution; (iii) self-manifestation; and (iv) self-expansion. Importantly, these different layers of the self can be characterized by different neuronal correlates—this results in different neuronally grounded configurations or organizations of personality. These layers correspond to different levels of personality organization, such as psychotic (as related to the layer of self-constitution), borderline (as related to the layer of self-manifestation) and neurotic (as related to the layer of self-expansion). Taken together, we provide here for the first time a neurobiologically and clinically grounded model of personality organization, which carries major psychodynamic and neuroscientific implications. The study of the spontaneous activity of the brain, intrinsically related to the self (rest–self overlap/containment) and the interaction with stimuli (rest–stimulus interaction) may represent a further advance in understanding how our *default* state plays a crucial role in navigating through the internal world and the external reality.

## Introduction: Self-Other Organization of Personality

Personality can be considered as a dimension or a continuum from healthy features, characterized by a coherent sense of the self and identity, engagements in satisfying relationships, relatively flexible functioning when stressed by external events or internal conflicts, appropriate expression of impulses and emotions, internalized moral values and maladaptive features. These are characterized by identity diffusion and incoherent sense of the self, problems in self-other differentiation and relatedness, lack or transient loss in reality testing, problems in affect and impulse regulation, mentalization and attention and inflexibility and rigidity in several domains (Kernberg and Caligor, 2005; Bateman and Fonagy, 2010; Kernberg, 2016; Lingiardi and McWilliams, 2017).

Historically several authors tried to classify patients who did not reach the criteria to be placed either in neurotic or in psychotic diagnosis (e.g., Knight, 1953). However, it was only in 1967 that Kernberg (1967) proposed a broader concept of Borderline personality organization (BPO), which included the evaluation of the identity (see also Erikson, 1968), of defense mechanisms and reality testing, as closely associated with the continuity and coherence of the sense of self and significant others. (Kohut (1971) conceptualized how a failure in the development of a cohesive sense of the self, depending from the interaction with the environment, leads to a fragmentation of the body, self, mind and the self-object. Recently, several authors, departing from the background of the attachment theory (Schore, 2000, 2001, 2012; Lyons-Ruth, 2003, 2008; Fonagy et al., 2007; Mucci, 2013, 2017; Beebe and Lachmann, 2014), proposed that the parent-infant dyad can be considered as the first intersubjective encounter that predisposes the development of the self and emphasized how the dual caregiver–infant exchange continuously modulates the formation of the growing subject, organizing the mind-body-brain interoceptive and exteroceptive connections in relation to the other.

Nowadays there are empirical evidences postulating that the degree of impairments in levels of self-definition and interpersonal relations are core features in defining the personality disorders (see Bender et al., 2011; Morey et al., 2011; DSM-5, American Psychiatric Association, 2013). The intrapsychic structure of the self–other personality organization is based on a neurobiological one (Kernberg, 2015).

## Neuroscientific Correlates of The Self and Its Relation with The Brain’S Spontaneous/Resting State Activity

The self has been investigated extensively in neuroscience and has been related to a cerebral network also recruited during the resting state, the other’s mind-reading, autobiographical memory, enhanced perception and embodied simulation (Metzinger and Gallese, 2003; Wicker et al., 2003; Gillihan and Farah, 2005; Northoff et al., 2006; Legrand and Ruby, 2009; Sui and Humphreys, 2015).

Among others, authors such as Northoff (Northoff and Bermpohl, 2004; Northoff et al., 2006; Northoff and Panksepp, 2008; Damasio, 2010, 2012; Panksepp and Biven, 2012) emphasized the existence of a complex, distributed and functionally based system of the self. The core self (Panksepp, 1998a,b) is considered as a trans-species functional entity based on subcortical midline structures (SCMS), in a mutually regulating process with the higher CMS, a more complex, reflective and conscious self-distributed sytem. This system allows the linking of the external events to the internal (motivational and emotional) impulses of the organism (Panksepp and Biven, 2012).

In addition to CMS and SCMS, a right lateralized frontoparietal network, including lateral somatosensory cortices overlapping with the distribution of mirror neuron areas, is also involved in self-recognition, self-awareness, social understanding and embodied simulation, i.e., in the re-enactment of sensory and motor experiences, empathy, mentalizing and symbolic activity (Gallese, 2007; Rizzolatti and Sinigaglia, 2008; Iacoboni, 2009; Keysers et al., 2010; Panksepp and Biven, 2012; Cozolino, 2014; Siegel, 2015).

Self-processing has been operationalized in many experimental studies in terms of self-relatedness (SR, Northoff, 2016a) and has been associated with the basic functions such as perception (Sui et al., 2012, 2013), action (Frings and Wentura, 2014), reward (de Greck et al., 2008) and emotions (Phan et al., 2004; Northoff et al., 2009).

Intriguingly, CMS, a core part of the Default Mode Network (DMN; Raichle et al., 2001; Buckner et al., 2008), has been associated with SR not only during the stimulus-induced states but also during the resting state characterized by spontaneous thought (Gusnard and Raichle, 2001; Zhu, 2004; D’Argembeau et al., 2005; Moran et al., 2006; Schneider et al., 2008; Enzi et al., 2009; Northoff et al., 2010; Whitfield-Gabrieli et al., 2011; Hu et al., 2016).

Therefore, the “rest-self overlap” concept (Bai et al., 2016; Northoff, 2016a) has been introduced to describe the convergence in anterior and posterior CMS (Qin and Northoff, 2011; Murray et al., 2015; Davey et al., 2016) between the self and the brain’s spontaneous (or resting state) activity.

Based on these findings one may hypothesize that the spontaneous activity of the brain may contain some specific information related to the self, serving to process and assign contents to the subsequent internal or external stimuli. Therefore, one may conceptualize a “rest-self overlap/containment” (Huang et al., 2016, 2017; Northoff, 2016a), where the self-specific information, not only overlap with the resting state, but are contained in the spontaneous activity and may provide the basis for the assignment of contents as processed in affective, cognitive, social and sensorimotor functions.

## Early Relational Experiences, Attachment and The Development of The Self from The Other

Early relational experiences and attachment play a fundamental role in shaping the sense of self, the sense of relatedness, and the capacities to regulate emotions and to mentalize, leading to what Bowlby (1969) has defined as internal working models (IWM). The relational internalization of benign or adverse interpersonal experiences is enabled by the human capacity for intersubjectivity, attunement and empathy, which are present from birth (Stern, 1985; Meltzoff and Brooks, 2001; Trevarthen, 2001; Meltzoff and Decety, 2003; Tronick, 2007).

Traumatic experiences, particularly attachment trauma and early relational adverse experiences, not only foster dissociation and psychopathology (Schimmenti, 2017) creating a vertical disconnection in the mind-brain-body system but also elicit the impulsivity, lack of effortful control, emotional dysregulation and use of immature defenses that are characteristics of self-other pathologies (see alternative model of personality disorders, DSM-5, 2013), such as BPO or maladaptive personality pathologies in general (Mucci, 2016, 2018; Granieri et al., 2017, 2018).

The enduring effect of early traumatization does not allow the connection between the limbic areas and superior orbito-frontal areas (Schore, 2000, 2001, 2003, 2012) creating the dysfunctions typical of personality pathologies, characterized by long-term abuse and dysfunctional families (Felitti et al., 1998; Mucci, 2013; Schimmenti and Caretti, 2016; Liotti, 2017; Scalabrini et al., 2017a).

Decety and Sommerville (2003) proposed how the distinct cognitive representations of self and others are related to self–other processing in the brain and how the right hemisphere plays a predominant role in the way that the self is connected to the other. In this regard, Schore has suggested that early relational trauma between mother and child alters the development of the right brain (Schore, 2001).

The early growth and maturation of brain regions involved in self and social development (Pfeifer and Peake, 2012) is experience-dependent and requires nurturing self-other interactions in the context of attachment for developing the capacity to regulate cognitive and emotional states (Messina et al., 2016b). For instance, a recent study (Brauer et al., 2016) indicated how 5-year old children exposed to higher maternal touch experiences show additional regional connectivity within the right dorso-medial prefrontal cortex and may benefit in terms of “social brain development.” In contrast, when an individual is denied these positive experiences, serious failures of self-development occur (Schore, 2005; Messina et al., 2016a; Mucci, 2016, 2018).

Recently, several studies investigated the neurobiology of attachment in animals (Insel and Young, 2001) and humans using functional magnetic resonance imaging (fMRI; Lorberbaum et al., 2002; Bartels and Zeki, 2004; Swain et al., 2007; Strathearn et al., 2008; Laurita et al., 2017, 2018). These studies showed how the regions prevalently located in the CMS and limbic areas are fundamentally involved in the context of attachment and have an impact on self-other related functions. Other studies investigated the neural correlates of attachment trauma, as in the case of borderline personality disorder (e.g., Buchheim et al., 2008).

**Figure 1:**
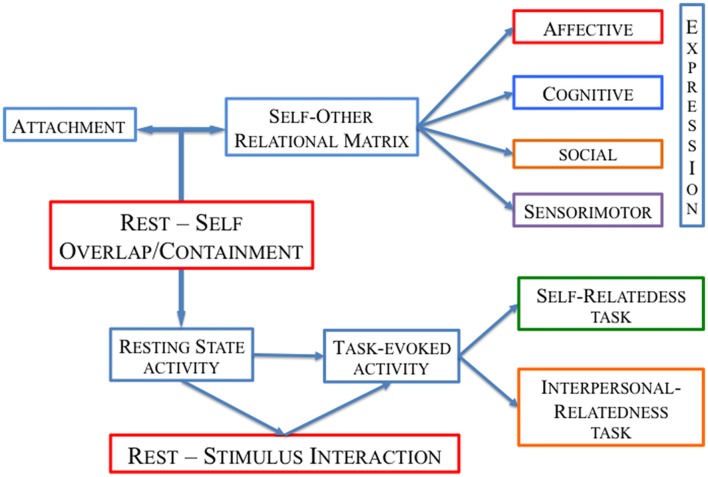
Schematic illustration for the study of the “Brain, Self and Personality”: the *Rest-Self overlap/containment* and *Rest-Stimulus interaction* model.

Departing from this background, if attachment influences the development of the self, the constitution, and the differentiation of the self and others and is considered closely linked to SR processing (Brockman, 2002; Northoff, 2011), we may expect an impact on the spontaneous/resting state activity of the brain, as suggested by a study showing a relation between increased measures of individual negative childhood experiences and a more entropic neuronal activity in medial prefrontal cortex during rest (Duncan et al., 2015; see Figure 1). Moreover, research on the resting state fMRI showed that emotional processing of attachment-related content induces carryover effects and alters the brain network configurations at rest (Krause et al., 2016, 2018; Borchardt et al., 2018).

## Neuropsychodynamic Model of Self and Personality Organization

Given the role of the experience-dependent (or attachment-dependent) rest-self overlap/containment, we hypothesize that self-specific information of individuals, and their personality features, may be present in the resting state activity and may shape task-evoked activity.

In summary, the study of personality needs to consider the so-called rest-stimulus interaction (Northoff et al., 2010) as a way to conceive how the brain’s intrinsic activity encodes self-specific information of the past and (possible) future input-output relationship (Northoff, 2016a).

**Figure 2:**
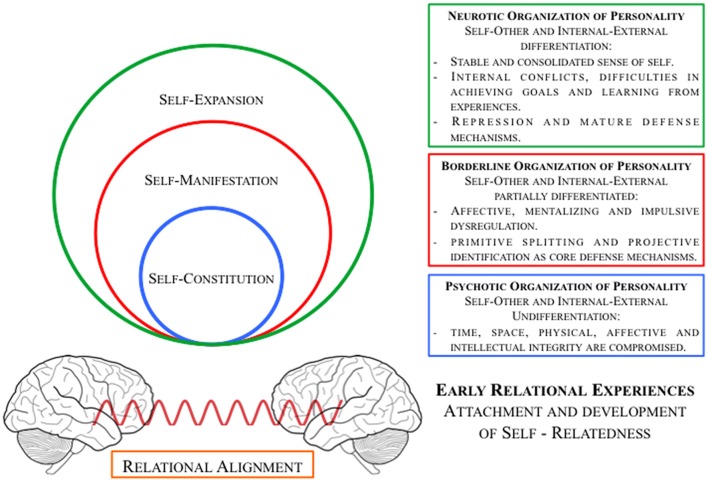
Schematic illustration of intrinsic neuronal and intrapsychic organization of self and personality: association of different *Rest-Self states* and *levels of personality organizations*.

In this context, we propose a novel conceptualization that aims to link the rest-self states and personality organization (see Figure 2).

In our view, different and interconnected states of the self are embedded in the intrinsic activity of the brain that predisposes the construction of subjectivity and consciousness (Northoff, 2017).

First, we propose that *Relational Alignment* is a prerequisite that gives the framework for the construction of the *Self*, a sort of neuro-ecological continuum between the brain and the external world. It is given by the first relational encounter with a caregiver and his/her capacity to attune with the mind-brain of the infant. The infant’s brain, if facilitated by a secure environment, starts becoming a part of the world’s time and space by the relational alignment with the temporo-spatial structure of the animate and inanimate reality (Schore, 2000, 2001; Trevarthen and Aitken, 2001; Pfeifer and Peake, 2012; Northoff, 2017). This predisposes the constitution and the development of the *Self*.

*Self-Constitution* represents the building blocks of the consciousness processing—it includes the construction (rather than perception) of both time and space. It is linked with the ownership of one’s own body, location of self in space, authorship and control of one’s own actions, and difference between fantasy and reality. It is linked with the capacity to distinguish the self from the non-self and internal from external. Thus, it is strongly connected with reality testing and it is the self-state that distinguishes the psychotic organization of personality from the others.

*Self-Manifestation* represents the actual consciousness in the present moment—it includes the experience of time and space, the perception of the environment, the affective and motivatinal system, the identification with social reality and cognitive functions such as thinking, imagining and mentalizing. It is particularly linked to the degree of integration of the self and the significant others. When the manifestation of the self oscillates between incoherent and/or disaggregated self-states we are navigating in the field of borderline and/or (early) trauma related pathologies.

*Self-Expansion* is linked with the stable and integrated aspects of the self in time and space: (a) autobiographical self, (b) social self, (c) linguistic self, and (d) mental self. It refers to the capacity to inhibit behaviors and to tolerate the ambivalence of the affects considering the past, present and future. The higher the capacity to self-expand and bind information in perception and memory (Sui and Humphreys, 2015), the higher the integration of various aspects of the self and others. A difficulty in self-expansion may be related to a difficulty in elaborating an external stimulus or an internal conflict as in neuroticism.

How are these different states of the self linked to the intra-psychic structure of individuals?

Since the brain’s intrinsic activity can be characterized by an individualized temporo-spatial structure, we suppose that all contents (whether affective, cognitive, social, or sensorimotor) and their underlying extrinsic activity must first and foremost be integrated within the brain’s spontaneous (internal) activity. The degree and the way the contents and their activities are integrated into the brain’s spontaneous activity determine how we perceive them and hence how we experience them, i.e., our subjective or self-conscious experience of reality (Northoff, 2016a,b,c; Northoff and Huang, 2017).

*Psychotic personality organization* (PPO) on a psychological level is characterized by identity diffusion, primitive defense mechanism, and loss of reality testing. On a neurobiological level we may find disturbances at a level of Self-Constitution, a disruption in the global organization of the brain’s intrinsic activity (Northoff, 2015).

The whole topography over all the networks and frequency ranges are disrupted (Rotarska-Jagiela et al., 2010; Shim et al., 2010; Khadka et al., 2013) and, for instance, the usual negative correlation between the DMN and the Control Executive Network (CEN), that are usually characterized by anticorrelation, are in psychosis transformed into a positive correlation which in turn may lead to the breakdown of the rest-self overlap where there is a self-assignment to either internal or external stimuli (Carhart-Harris et al., 2012, 2013).

In this case there is no possibility to differentiate the internal world from the external reality. The relationship from the CMS and the somatosensory network is altered, ending up in a lack of differentiation in processing the intrinsic and extrinsic stimuli (Ebisch and Aleman, 2016; Northoff and Duncan, 2016), which results in identity diffusion or fragmentation of Self-constitution. We may hypothesize that this psychotic organization shows severe impairments at a pre-phenomenal level of experience on the spatiotemporal structure of the brain’s intrinsic activity (Northoff, 2016b,c).

*BPO* is characterized by intact reality testing, primitive defense mechanism based on splitting and projective identification and identity diffusion. At a neurobiological level we hypothesize that the whole brain topography and organization between the networks are partially preserved but the balance between them consequently shows abnormalities, as in the case of bipolar disorder (Magioncalda et al., 2015; Martino et al., 2016), and that the subsequent relation with the external stimuli may be impaired. Indeed, a recent meta-analysis (Visintin et al., 2016) shows increased activity in the regions spanning across the midline core of DMN in patients with borderline personality disorder during the resting state, which may imply difficulties in self-referential, social and emotional processing (Van Overwalle, 2009; Etkin et al., 2011).

We may find abnormalities in the rest-stimulus interaction (e.g., in narcissistic personality features; Scalabrini et al., 2017b) and lack of integration in the brain’s self-other networks (Herpertz et al., 2018): for example, in borderline personality disorders we can observe alterations in the orbitofrontal cortex and the connected subcortical regions (Minzenberg et al., 2007; Koenigsberg et al., 2009; Enzi et al., 2013). Moreover, other studies have described the functional neuroanatomy of borderline disorders that are associated with the hypersensitivity, intolerance for aloneness, and attachment fears typical of patients in this broad diagnostic group (Buchheim et al., 2008; King-Casas et al., 2008; Fertuck et al., 2009; Dziobek et al., 2011).

We may hypothesize that individuals with BPO show severe impairments at a pre-reflective level of experience, at a Self-manifestation state, where the implicit encoded information (Mucci, 2016) about the experiences of self, body, and others are related to the spatiotemporal structure of the brain’s intrinsic activity.

*Neurotic personality organization (NPO)* is characterized by intact reality testing, mature defense mechanism based on repression, and integrated sense of self and identity. In NPO we may find impairments at the Self-Expansion state, where at a neurobiological level we hypothesize that the whole brain’s topography, organization between networks and balance between them are preserved but their coherence is not given for granted. We may expect to find a decreased coherence within the networks and decreased cross-frequency coupling while the spatiotemporal structure by itself is well integrated. In detail, we hypothesize that the measures such as functional connectivity provide no information on the intrinsic neuronal activity (Lu and Stein, 2014; Raichle, 2015), while the measures such as regional of homogeneity (ReHo), low frequency oscillation (LFO), and measures of complexity (e.g., Power Law exponent, H-Hurst) are more related to the intrinsic local brain activity, so that they may be more fine-grained to detect the trait-features of personality, as in the case of neuroticism. Studies investigating neuroticism in the resting state fMRI found that the ReHo in the prefrontal cortex was negatively modulated by the neurotic personality features (Wei et al., 2011; Gentili et al., 2017), thereby supporting the hypothesis of neurotic DMN alterations in the resting state analysis, as well as during task (Wei et al., 2011, 2016; Forbes et al., 2014; Sampaio et al., 2014; Tzschoppe et al., 2014).

Thus, NPO individuals have difficulty to expand and finalize their selves over time because of their internal conflicts. We may hypothesize that a certain incoherence in the brain functioning is related to the difficulty of these individuals in processing certain contents at the reflective level of the experience (explicit experience of cognitive, affective, social and sensorimotor functions).

## Conclusion

Neuroscience has considerably advanced in revealing the neural correlates of the self, which are closely related to the cortical midline structure and their spontaneous activity. However, the relationship of self to its personality, as discussed in psychoanalysis and psychiatry, still remains to be further elucidated. The present article is a first attempt to bridge this gap. Based on the recent findings, we propose here a multi-layered neuronally-based model of the self with: (i) relational alignment; (ii) self-constitution; (iii) self-manifestation; and (iv) self-expansion. We suggest that these layers of self correspond to the different levels of personality organization including psychotic, borderline and neurotic. These levels can be distinguished from each other through their neuronal and defense mechanisms.

This amounts to a novel neuropsychodynamic model of personality organization that bridges the gap between self, as dealt with in neuroscience and psychology, and personality as conceptualized in psychoanalysis.

Our perspective opens not only novel doors to our understanding of the personality and its alterations but also novel forms of therapeutic intervention, due to the neuronal basis in spontaneous activity of the brain. Therapeutic interventions need to achieve, first and foremost, a relational alignment between the therapist and the patient in order to lead the self to expand and be more connected to the internal world and external reality.

## Author Contributions

AS, CM and GN wrote the article.

## Conflict of Interest Statement

The authors declare that the research was conducted in the absence of any commercial or financial relationships that could be construed as a potential conflict of interest.

## References

[B1] American Psychiatric Association (2013). Diagnostic and Statistical Manual of Mental Disorders (DSM-5^®^). Washington, DC: American Psychiatric Association.

[B2] Bai Y. Nakao T. Xu J. Qin P. Chaves P. Heinzel A.. (2016). Resting state glutamate predicts elevated pre-stimulus α during self-relatedness: a combined EEG-MRS study on “rest-self overlap”. Soc. Neurosci. 11, 249–263. 10.1080/17470919.2015.1072582 26207415

[B3] Bartels A. Zeki S. (2004). The neural correlates of maternal and romantic love. Neuroimage 21, 1155–1166. 10.1016/j.neuroimage.2003.11.003 15006682

[B4] Bateman A. Fonagy P. (2010). Mentalization based treatment for borderline personality disorder. World Psychiatry 9, 11–15. 10.1002/j.2051-5545.2010.tb00255.x 20148147 PMC2816926

[B5] Beebe B. Lachmann F. M. (2014). The Origins of Attachment: Infant Research and Adult Treatment. New York, NY: Routledge.

[B6] Bender D. S. Morey L. C. Skodol A. E. (2011). Toward a model for assessing level of personality functioning in DSM-5, Part I: a review of theory and methods. J. Pers. Assess. 93, 332–346. 10.1080/00223891.2011.583808 22804672

[B7] Borchardt V. Surova G. van der Meer J. Bola M. Frommer J. Leutritz A. L.. (2018). Exposure to attachment narratives dynamically modulates cortical arousal during the resting state in the listener. Brain Behav. 8:e01007. 10.1002/brb3.1007 29877060 PMC6043700

[B8] Bowlby J. (1969). Attachment and Loss, Vol.1: Attachment. New York, NY: Basic Books.

[B203] Brauer J. Xiao Y. Poulain T. Friederici A. D. Schirmer A. (2016). Frequency of maternal touch predicts resting activity and connectivity of the developing social brain. Cereb. Cortex 26, 3544–3552. 10.1093/cercor/bhw137 27230216 PMC4961023

[B9] Brockman R. (2002). Self, object, neurobiology. Neuropsychoanalysis 4, 89–101. 10.1080/15294145.2002.10773382

[B10] Buchheim A. Erk S. George C. Kächele H. Kircher T. Martius P.. (2008). Neural correlates of attachment trauma in borderline personality disorder: a functional magnetic resonance imaging study. Psychiatry Res. 163, 223–235. 10.1016/j.pscychresns.2007.07.001 18635342

[B11] Buckner R. L. Andrews-Hanna J. R. Schacter D. L. (2008). The brain’s default network. Ann. N Y Acad. Sci. 1124, 1–38. 10.1196/annals.1440.011 18400922

[B204] Carhart-Harris R. L. Brugger S. Nutt D. J. Stone J. M. (2013). Psychiatry’s next top model: cause for a re-think on drug models of psychosis and other psychiatric disorders. J. Psychopharmacol. 27, 771–778. 10.1177/0269881113494107 23784738

[B205] Carhart-Harris R. L. Leech R. Erritzoe D. Williams T. M. Stone J. M. Evans J.. (2012). Functional connectivity measures after psilocybin inform a novel hypothesis of early psychosis. Schizophr. Bull. 39, 1343–1351. 10.1093/schbul/sbs117 23044373 PMC3796071

[B12] Cozolino L. (2014). The Neuroscience of Human Relationships: Attachment and the Developing Social Brain. 2nd Edn. New York, NY: W.W. Norton & Company.

[B15] Damasio A. R. (2010). Self Comes to Mind: Constructing the Conscious Brain. New York, NY: Random House.

[B14] Damasio A. R. (2012). Neuroscience and psychoanalysis: a natural alliance. Psychoanal. Rev. 99, 591–594. 10.1521/prev.2012.99.4.591

[B16] D’Argembeau A. Collette F. Van der Linden M. Laureys S. Del Fiore G. Degueldre C.. (2005). Self-referential reflective activity and its relationship with rest: a PET study. Neuroimage 25, 616–624. 10.1016/j.neuroimage.2004.11.048 15784441

[B17] Davey C. G. Pujol J. Harrison B. J. (2016). Mapping the self in the brain’s default mode network. Neuroimage 132, 390–397. 10.1016/j.neuroimage.2016.02.022 26892855

[B18] de Greck M. Rotte M. Paus R. Moritz D. Thiemann R. Proesch U.. (2008). Is our self based on reward? Self-relatedness recruits neural activity in the reward system. Neuroimage 39, 2066–2075. 10.1016/j.neuroimage.2007.11.006 18155927

[B19] Decety J. Sommerville J. A. (2003). Shared representations between self and other: a social cognitive neuroscience view. Trends Cogn. Sci. 7, 527–533. 10.1016/j.tics.2003.10.004 14643368

[B20] Duncan N. W. Hayes D. J. Wiebking C. Tiret B. Pietruska K. Chen D. Q.. (2015). Negative childhood experiences alter a prefrontal-insular-motor cortical network in healthy adults: a preliminary multimodal rsfMRI-fMRI-MRS-dMRI study. Hum. Brain Mapp. 36, 4622–4637. 10.1002/hbm.22941 26287448 PMC4827445

[B21] Dziobek I. Preißler S. Grozdanovic Z. Heuser I. Heekeren H. R. Roepke S. (2011). Neuronal correlates of altered empathy and social cognition in borderline personality disorder. Neuroimage 57, 539–548. 10.1016/j.neuroimage.2011.05.005 21586330

[B22] Ebisch S. J. Aleman A. (2016). The fragmented self: imbalance between intrinsic and extrinsic self-networks in psychotic disorders. Lancet Psychiatry 3, 784–790. 10.1016/s2215-0366(16)00045-6 27374147

[B23] Enzi B. De Greck M. Pröesch U. Tempelmann C. Northoff G. (2009). Is our self nothing but reward? Neuronal overlap and distinction between reward and personal relevance and its relation to human personality. PLoS One 4:e8429. 10.1371/journal.pone.0008429 20041155 PMC2794541

[B24] Enzi B. Doering S. Faber C. Hinrichs J. Bahmer J. Northoff G. (2013). Reduced deactivation in reward circuitry and midline structures during emotion processing in borderline personality disorder. World J. Biol. Psychiatry 14, 45–56. 10.3109/15622975.2011.579162 21732733

[B25] Erikson E. H. (1968). Psychoanalysis and theories of man. (Book reviews: identity: youth and crisis; Childhood and society (1950). Science 161, 257–258.

[B26] Etkin A. Egner T. Kalisch R. (2011). Emotional processing in anterior cingulate and medial prefrontal cortex. Trends Cogn. Sci. 15, 85–93. 10.1016/j.tics.2010.11.004 21167765 PMC3035157

[B27] Felitti V. J. Anda R. F. Nordenberg D. Williamson D. F. Spitz A. M. Edwards V.. (1998). Relationship of childhood abuse and household dysfunction to many of the leading causes of death in adults: the Adverse Childhood Experiences (ACE) Study. Am. J. Prev. Med. 14, 245–258. 10.1016/S0749-3797(98)00017-8 9635069

[B28] Fertuck E. A. Jekal A. Song I. Wyman B. Morris M. C. Wilson S. T.. (2009). Enhanced ‘reading the mind in the eyes’ in borderline personality disorder compared to healthy controls. Psychol. Med. 39, 1979–1988. 10.1017/S003329170900600X 19460187 PMC3427787

[B29] Fonagy P. Gergely G. Target M. (2007). The parent-infant dyad and the construction of the subjective self. J. Child Psychol. Psychiatry 48, 288–328. 10.1111/j.1469-7610.2007.01727.x 17355400

[B30] Forbes C. E. Poore J. C. Krueger F. Barbey A. K. Solomon J. Grafman J. (2014). The role of executive function and the dorsolateral prefrontal cortex in the expression of neuroticism and conscientiousness. Soc. Neurosci. 9, 139–151. 10.1080/17470919.2013.871333 24405294

[B31] Frings C. Wentura D. (2014). Self-priorization processes in action and perception. J. Exp. Psychol. Hum. Percept. Perform. 40, 1737–1740. 10.1037/a0037376 24999614

[B32] Gallese V. (2007). Before and below ‘theory of mind’: embodied simulation and the neural correlates of social cognition. Philos. Trans. R. Soc. Lond. B Biol. Sci. 362, 659–669. 10.1098/rstb.2006.2002 17301027 PMC2346524

[B33] Gentili C. Cristea I. A. Ricciardi E. Vanello N. Popita C. David D.. (2017). Not in one metric: neuroticism modulates different resting state metrics within distinctive brain regions. Behav. Brain Res. 327, 34–43. 10.1016/j.bbr.2017.03.031 28342970

[B34] Gillihan S. J. Farah M. J. (2005). Is self special? A critical review of evidence from experimental psychology and cognitive neuroscience. Psychol. Bull. 131, 76–97. 10.1037/0033-2909.131.1.76 15631554

[B35] Granieri A. Guglielmucci F. Costanzo A. Caretti V. Schimmenti A. (2018). Trauma-related dissociation is linked with maladaptive personality functioning. Front. Psychiatry 9:206. 10.3389/fpsyt.2018.00206 29887807 PMC5980986

[B36] Granieri A. La Marca L. Mannino G. Giunta S. Guglielmucci F. Schimmenti A. (2017). The relationship between defense patterns and DSM-5 maladaptive personality domains. Front. Psychol. 8:1926. 10.3389/fpsyg.2017.01926 29163301 PMC5673655

[B37] Gusnard D. A. Raichle M. E. (2001). Searching for a baseline: functional imaging and the resting human brain. Nat. Rev. Neurosci. 2, 685–694. 10.1038/35094500 11584306

[B38] Herpertz S. C. Bertsch K. Jeung H. (2018). Neurobiology of criterion a: self and interpersonal personality functioning. Curr. Opin. Psychol. 21, 23–27. 10.1016/j.copsyc.2017.08.032 28946053

[B39] Hu C. Di X. Eickhoff S. B. Zhang M. Peng K. Guo H.. (2016). Distinct and common aspects of physical and psychological self-representation in the brain: a meta-analysis of self-bias in facial and self-referential judgements. Neurosci. Biobehav. Rev. 61, 197–207. 10.1016/j.neubiorev.2015.12.003 26695384

[B40] Huang Z. Obara N. Davis H. H. IV. Pokorny J. Northoff G. (2016). The temporal structure of resting-state brain activity in the medial prefrontal cortex predicts self-consciousness. Neuropsychologia 82, 161–170. 10.1016/j.neuropsychologia.2016.01.025 26805557

[B41] Huang Z. Zhang J. Longtin A. Dumont G. Duncan N. W. Pokorny J.. (2017). Is there a nonadditive interaction between spontaneous and evoked activity? Phase-dependence and its relation to the temporal structure of scale-free brain activity. Cereb. Cortex 27, 1037–1059. 10.1093/cercor/bhv288 26643354

[B42] Iacoboni M. (2009). Mirroring People: The New Science of How we Connect with Others. New York, NY: Macmillan.

[B43] Insel T. R. Young L. J. (2001). The neurobiology of attachment. Nat. Rev. Neurosci. 2, 129–136. 10.1038/35053579 11252992

[B44] Kernberg O. F. (1967). Borderline personality organization. J. Am. Psychoanal. Assoc. 15, 641–685. 10.1177/000306516701500309 4861171

[B45] Kernberg O. F. (2015). Neurobiological correlates of object relations theory: the relationship between neurobiological and psychodynamic development. Int. Forum Psychoanal. 24, 38–46. 10.1080/0803706X.2014.912352

[B46] Kernberg O. F. (2016). What is personality? J. Pers. Disord. 30, 145–156. 10.1521/pedi.2106.30.2.145 27027422

[B47] Kernberg O. F. Caligor E. (2005). “A psychoanalytic theory of personality disorders,” in Major Theories of Personality Disorders, 2nd Edn. eds Clarkin J. F. Lenzenweger M. F. (New York, NY: Guilford Press), 114–156.

[B48] Keysers C. Kaas J. H. Gazzola V. (2010). Somatosensation in social perception. Nat. Rev. Neurosci. 11, 417–428. 10.1038/nrn2833 20445542

[B49] Khadka S. Meda S. A. Stevens M. C. Glahn D. C. Calhoun V. D. Sweeney J. A.. (2013). Is aberrant functional connectivity a psychosis endophenotype? A resting state functional magnetic resonance imaging study. Biol. Psychiatry 74, 458–466. 10.1016/j.biopsych.2013.04.024 23746539 PMC3752322

[B50] King-Casas B. Sharp C. Lomax-Bream L. Lohrenz T. Fonagy P. Montague P. R. (2008). The rupture and repair of cooperation in borderline personality disorder. Science 321, 806–810. 10.1126/science.1156902 18687957 PMC4105006

[B51] Knight R. P. (1953). Borderline states. Bull. Menninger Clin. 17, 1–12. 13009379

[B52] Koenigsberg H. W. Siever L. J. Lee H. Pizzarello S. New A. S. Goodman M.. (2009). Neural correlates of emotion processing in borderline personality disorder. Psychiatry Res. 172, 192–199. 10.1016/j.pscychresns.2008.07.010 19394205 PMC4153735

[B53] Kohut H. (1971). The Analysis of the Self. New York, NY: International Universities Press.

[B54] Krause A. L. Borchardt V. Li M. van Tol M. J. Demenescu L. R. Strauss B.. (2016). Dismissing attachment characteristics dynamically modulate brain networks subserving social aversion. Front. Hum. Neurosci. 10:77. 10.3389/fnhum.2016.00077 27014016 PMC4783398

[B55] Krause A. L. Colic L. Borchardt V. Li M. Strauss B. Buchheim A.. (2018). Functional connectivity changes following interpersonal reactivity. Hum. Brain Mapp. 39, 866–879. 10.1002/hbm.23888 29164726 PMC6866275

[B56] Laurita A. C. Hazan C. Spreng R. N. (2017). Dissociable patterns of brain activity for mentalizing about known others: a role for attachment. Soc. Cogn. Affect. Neurosci. 12, 1072–1082. 10.1093/scan/nsx040 28407150 PMC5490684

[B57] Laurita A. C. Hazan C. Spreng R. N. (2018). Neural signatures of chronic accessibility in parent—adult child attachment bonds. Soc. Neurosci. [Epub ahead of print]. 10.1080/17470919.2018.1494037 29949456

[B58] Legrand D. Ruby P. (2009). What is self-specific? Theoretical investigation and critical reviewof neuroimaging results. Psychol. Rev. 116, 252–282. 10.1037/a0014172 19159156

[B59] Lingiardi V. McWilliams N. (2017). Psychodynamic Diagnostic Manual. Second Edition (PDM-2). New York, NY: Guilford Press. 10.1002/wps.20233 PMC4471982 26043343

[B60] Liotti G. (2017). Conflicts between motivational systems related to attachment trauma: key to understanding the intra-family relationship between abused children and their abusers. J. Trauma Dissociation 18, 304–318. 10.1080/15299732.2017.1295392 28318416

[B61] Lorberbaum J. P. Newman J. D. Horwitz A. R. Dubno J. R. Lydiard R. B. Hamner M. B.. (2002). A potential role for thalamocingulate circuitry in human maternal behavior. Biol. Psychiatry 51, 431–445. 10.1016/s0006-3223(01)01284-7 11922877

[B62] Lu H. Stein E. A. (2014). Resting state functional connectivity: its physiological basis and application in neuropharmacology. Neuropharmacology 84, 79–89. 10.1016/j.neuropharm.2013.08.023 24012656

[B63] Lyons-Ruth K. (2003). Dissociation and the parent-infant dialogue: a longitudinal perspective from attachment research. J. Am. Psychoanal. Assoc. 51, 883–911. 10.1177/00030651030510031501 14596565

[B64] Lyons-Ruth K. (2008). Contributions of the mother-infant relationship to dissociative, borderline, and conduct symptoms in young adulthood. Infant Ment. Health J. 29, 203–218. 10.1002/imhj.20173 19122769 PMC2613366

[B65] Magioncalda P. Martino M. Conio B. Escelsior A. Piaggio N. Presta A.. (2015). Functional connectivity and neuronal variability of resting state activity in bipolar disorder—reduction and decoupling in anterior cortical midline structures. Hum. Brain Mapp. 36, 666–682. 10.1002/hbm.22655 25307723 PMC6869107

[B66] Martino M. Magioncalda P. Huang Z. Conio B. Piaggio N. Duncan N. W.. (2016). Contrasting variability patterns in the default mode and sensorimotor networks balance in bipolar depression and mania. Proc. Natl. Acad. Sci. U S A 113, 4824–4829. 10.1073/pnas.1517558113 27071087 PMC4855585

[B67] Meltzoff A. N. Brooks R. (2001). ““Like me” as a building block for understanding other minds: bodily acts, attention, and intention,” in Intentions and Intentionality: Foundations of Social Cognition, eds Malle B. F. Moses L. J. Baldwin D. A. (Cambridge, MA: The MIT Press), 171–191.

[B68] Meltzoff A. N. Decety J. (2003). What imitation tells us about social cognition: a rapprochement between developmental psychology and cognitive neuroscience. Philos. Trans. R. Soc. Lond. B Biol. Sci. 358, 491–500. 10.1098/rstb.2002.1261 12689375 PMC1351349

[B69] Messina I. Bianco F. Cusinato M. Calvo V. Sambin M. (2016a). Abnormal default system functioning in depression: implications for emotion regulation. Front. Psychol. 7:858. 10.3389/fpsyg.2016.00858 27375536 PMC4901076

[B70] Messina I. Sambin M. Beschoner P. Viviani R. (2016b). Changing views of emotion regulation and neurobiological models of the mechanism of action of psychotherapy. Cogn. Affect. Behav. Neurosci. 16, 571–587. 10.3758/s13415-016-0440-5 27351671

[B71] Metzinger T. Gallese V. (2003). The emergence of a shared action ontology: building blocks for a theory. Conscious. Cogn. 12, 549–571. 10.1016/s1053-8100(03)00072-2 14656499

[B72] Minzenberg M. J. Fan J. New A. S. Tang C. Y. Siever L. J. (2007). Fronto-limbic dysfunction in response to facial emotion in borderline personality disorder: an event-related fMRI study. Psychiatry Res. 155, 231–243. 10.1016/j.pscychresns.2007.03.006 17601709 PMC2084368

[B73] Moran J. M. Macrae C. N. Heatherton T. F. Wyland C. L. Kelley W. M. (2006). Neuroanatomical evidence for distinct cognitive and affective components of self. J. Cogn. Neurosci. 18, 1586–1594. 10.1162/jocn.2006.18.9.1586 16989558

[B74] Morey L. C. Berghuis H. Bender D. S. Verheul R. Krueger R. F. Skodol A. E. (2011). Toward a model for assessing level of personality functioning in DSM-5, Part II: empirical articulation of a core dimension of personality pathology. J. Pers. Assess. 93, 347–353. 10.1080/00223891.2011.577853 22804673

[B75] Mucci C. (2013). Beyond Individual and Collective Trauma. Intergenerational Transmission, Psychoanalytic Treatment, and the Dynamics of Forgiveness. London: Karnac Books.

[B76] Mucci C. (2016). “Implicit memory, unrepressed uncosncious, and trauma theory: the turn of the screw between contemporary psychoanalysis and neuroscence,” in Unrepressed Unconscious, Implicit Memory and Clinical Work, eds Craparo G. Mucci C. (London: Karnac Books), 99–129.

[B77] Mucci C. (2017). Ferenczi’s revolutionary therapeutic approach. Am. J. Psychoanal. 77, 239–254. 10.1057/s11231-017-9104-7 28751659

[B78] Mucci C. (2018). Borderline Bodies. Affect Regulation Therapy for Personality Disorders. New York, NY: W.W. Norton & Company.

[B202] Murray R. J. Debbane M. Fox P. T. Bzdok D. Eickhoff S. B. (2015). Functional connectivity mapping of regions associated with self- and other-processing. Hum. Brain Mapp. 36, 1304–1324. 10.1002/hbm.22703 25482016 PMC4791034

[B79] Northoff G. (2011). Neuropsychoanalysis in Practice: Brain, Self and Objects. New York, NY: Oxford University Press.

[B80] Northoff G. (2015). Is schizophrenia a spatiotemporal disorder of the brain’s resting state? World Psychiatry 14, 34–35. 10.1002/wps.20177 25655148 PMC4329887

[B81] Northoff G. (2016a). Is the self a higher-order or fundamental function of the brain? The “basis model of self-specificity” and its encoding by the brain’s spontaneous activity. Cogn. Neurosci. 7, 203–222. 10.1080/17588928.2015.1111868 26505808

[B82] Northoff G. (2016b). Spatiotemporal psychopathology I: no rest for the brain’s resting state activity in depression? Spatiotemporal psychopathology of depressive symptoms. J. Affect. Disord. 190, 854–866. 10.1016/j.jad.2015.05.007 26048657

[B83] Northoff G. (2016c). Spatiotemporal psychopathology II: how does a psychopathology of the brain’s resting state look like? Spatiotemporal approach and the history of psychopathology. J. Affect. Disord. 190, 867–879. 10.1016/j.jad.2015.05.008 26071797

[B84] Northoff G. (2017). Personal identity and cortical midline structure (CMS): do temporal features of CMS neural activity transform into “self-continuity”? Psychol. Inq. 28, 122–131. 10.1080/1047840x.2017.1337396

[B85] Northoff G. Bermpohl F. (2004). Cortical midline structures and the self. Trends Cogn. Sci. 8, 102–107. 10.1016/j.tics.2004.01.004 15301749

[B86] Northoff G. Duncan N. W. (2016). How do abnormalities in the brain’s spontaneous activity translate into symptoms in schizophrenia? From an overview of resting state activity findings to a proposed spatiotemporal psychopathology. Prog. Neurobiol. 145–146, 26–45. 10.1016/j.pneurobio.2016.08.003 27531135

[B89] Northoff G. Heinzel A. de Greck M. Bermpohl F. Dobrowolny H. Panksepp J. (2006). Self-referential processing in our brain—a meta-analysis of imaging studies on the self. Neuroimage 31, 440–457. 10.1016/j.neuroimage.2005.12.002 16466680

[B87] Northoff G. Huang Z. (2017). How do the brain’s time and space mediate consciousness and its different dimensions? Temporo-spatial theory of consciousness (TTC). Neurosci. Biobehav. Rev. 80, 630–645. 10.1016/j.neubiorev.2017.07.013 28760626

[B88] Northoff G. Panksepp J. (2008). The trans-species concept of self and the subcortical-cortical midline system. Trends Cogn. Sci. 12, 259–264. 10.1016/j.tics.2008.04.007 18555737

[B90] Northoff G. Qin P. Nakao T. (2010). Rest-stimulus interaction in the brain: a review. Trends Neurosci. 33, 277–284. 10.1016/j.tins.2010.02.006 20226543

[B91] Northoff G. Schneider F. Rotte M. Matthiae C. Tempelmann C. Wiebking C.. (2009). Differential parametric modulation of self-relatedness and emotions in different brain regions. Hum. Brain Mapp. 30, 369–382. 10.1002/hbm.20510 18064583 PMC6870760

[B92] Panksepp J. (1998a). The periconscious substrates of consciousness: affective states and the evolutionary origins of the SELF. J. Conscious. Stud. 5, 566–582.

[B93] Panksepp J. (1998b). Affective Neuroscience: The Foundations of Human and Animal Emotions. New York, NY: Oxford University Press.

[B94] Panksepp J. Biven L. (2012). The Archaeology of Mind: Neuroevolutionary Origins of Human Emotions. New York, NY: W. W. Norton & Company.

[B95] Pfeifer J. H. Peake S. J. (2012). Self-development: integrating cognitive, socioemotional, and neuroimaging perspectives. Dev. Cogn. Neurosci. 2, 55–69. 10.1016/j.dcn.2011.07.012 22682728 PMC6987679

[B96] Phan K. L. Taylor S. F. Welsh R. C. Ho S. H. Britton J. C. Liberzon I. (2004). Neural correlates of individual ratings of emotional salience: a trial-related fMRI study. Neuroimage 21, 768–780. 10.1016/j.neuroimage.2003.09.072 14980580

[B97] Qin P. Northoff G. (2011). How is our self related to midline regions and the default-mode network? Neuroimage 57, 1221–1233. 10.1016/j.neuroimage.2011.05.028 21609772

[B98] Raichle M. E. (2015). The restless brain: how intrinsic activity organizes brain function. Philos. Trans. R. Soc. Lond. B Biol. Sci. 370:20140172. 10.1098/rstb.2014.0172 25823869 PMC4387513

[B99] Raichle M. E. MacLeod A. M. Snyder A. Z. Powers W. J. Gusnard D. A. Shulman G. L. (2001). A default mode of brain function. Proc. Natl. Acad. Sci. U S A 98, 676–682. 10.1073/pnas.98.2.676 11209064 PMC14647

[B100] Rizzolatti G. Sinigaglia C. (2008). Mirrors in the Brain: How Our Minds Share Actions and Emotions. Oxford: Oxford University Press.

[B101] Rotarska-Jagiela A. van de Ven V. Oertel-Knöchel V. Uhlhaas P. J. Vogeley K. Linden D. E. (2010). Resting-state functional network correlates of psychotic symptoms in schizophrenia. Schizophr. Res. 117, 21–30. 10.1016/j.schres.2010.01.001 20097544

[B102] Sampaio A. Soares J. M. Coutinho J. Sousa N. Gonçalves Ó. F. (2014). The Big Five default brain: functional evidence. Brain Struct. Funct. 219, 1913–1922. 10.1007/s00429-013-0610-y 23881294

[B103] Scalabrini A. Cavicchioli M. Fossati A. Maffei C. (2017a). The extent of dissociation in borderline personality disorder: a meta-analytic review. J. Trauma Dissociation 18, 522–543. 10.1080/15299732.2016.1240738 27681284

[B104] Scalabrini A. Huang Z. Mucci C. Perrucci M. G. Ferretti A. Fossati A.. (2017b). How spontaneous brain activity and narcissistic features shape social interaction. Sci. Rep. 7:9986. 10.1038/s41598-017-10389-9 28855682 PMC5577167

[B105] Schimmenti A. (2017). The trauma factor: examining the relationships among different types of trauma, dissociation and psychopathology. J. Trauma Dissociation 1–20. 10.1080/15299732.2017.1402400 29125800

[B106] Schimmenti A. Caretti V. (2016). Linking the overwhelming with the unbearable: developmental trauma, dissociation, and the disconnected self. Psychoanal. Psychol. 33, 106–128. 10.1037/a0038019

[B107] Schneider F. Bermpohl F. Heinzel A. Rotte M. Walter M. Tempelmann C.. (2008). The resting brain and our self: self-relatedness modulates resting state neural activity in cortical midline structures. Neuroscience 157, 120–131. 10.1016/j.neuroscience.2008.08.014 18793699

[B108] Schore A. N. (2000). Attachment and the regulation of the right brain. Attach. Hum. Dev. 2, 23–47. 10.1080/146167300361309 11707891

[B109] Schore A. N. (2001). Effects of a secure attachment relationship on right brain development, affect regulation, and infant mental health. Infant Ment. Health J. 22, 7–66. 10.1002/1097-0355(200101/04)22:1<7::aid-imhj2>3.0.co;2-n

[B110] Schore A. N. (2003). Affect Dysregulation and Disorders of the Self (Norton Series on Interpersonal Neurobiology). New York, NY: W. W. Norton & Company.

[B111] Schore A. N. (2005). Attachment, affect regulation, and the developing right brain: linking developmental neuroscience to pediatrics. Pediatr. Rev. 26, 204–217. 10.1542/pir.26-6-204 15930328

[B112] Schore A. N. (2012). The Science of the Art of Psychotherapy (Norton Series on Interpersonal Neurobiology). New York, NY: W.W. Norton & Company.

[B113] Shim G. Oh J. S. Jung W. H. Jang J. H. Choi C. H. Kim E.. (2010). Altered resting-state connectivity in subjects at ultra-high risk for psychosis: an fMRI study. Behav. Brain Funct. 6:58. 10.1186/1744-9081-6-58 20932348 PMC2959003

[B114] Siegel D. J. (2015). The Developing Mind: How Relationships and The Brain Interact to Shape Who We Are. 2nd Edn. New York, NY: Guilford Publications.

[B115] Stern D. N. (1985). The Interpersonal World of the Infant: A View from Psychoanalysis and Developmental Psychology. London: Karnac Books.

[B116] Strathearn L. Li J. Fonagy P. Montague P. R. (2008). What’s in a smile? Maternal brain responses to infant facial cues. Pediatrics 122, 40–51. 10.1542/peds.2007-1566 18595985 PMC2597649

[B118] Sui J. Chechlacz M. Humphreys G. W. (2012). Dividing the self: distinct neural substrates of task-based and automatic self-prioritization after brain damage. Cognition 122, 150–162. 10.1016/j.cognition.2011.10.008 22115024

[B117] Sui J. Humphreys G. W. (2015). The integrative self: how self-reference integrates perception and memory. Trends Cogn. Sci. 19, 719–728. 10.1016/j.tics.2015.08.015 26447060

[B119] Sui J. Liu M. Mevorach C. Humphreys G. W. (2015). The salient self: the left intraparietal sulcus responds to social as well as perceptual-salience after self-association. Cereb. Cortex 25, 1060–1068. 10.1093/cercor/bht302 24165832

[B201] Sui J. Rotshtein P. Humphreys G. W. (2013). Coupling social attention to the self forms a network for personal significance. Proc. Natl. Acad. Sci. U. S. A. 110, 7607–7612. 10.1073/pnas.1221862110 23610386 PMC3651422

[B120] Swain J. E. Lorberbaum J. P. Kose S. Strathearn L. (2007). Brain basis of early parent-infant interactions: psychology, physiology and *in vivo* functional neuroimaging studies. J. Child Psychol. Psychiatry 48, 262–287. 10.1111/j.1469-7610.2007.01731.x 17355399 PMC4318551

[B122] Trevarthen C. (2001). Intrinsic motives for companionship in understanding: their origin, development, and significance for infant mental health. Infant Ment. Health J. 22, 95–131. 10.1002/1097-0355(200101/04)22:1<95::aid-imhj4>3.0.co;2-6

[B121] Trevarthen C. Aitken K. J. (2001). Infant intersubjectivity: research, theory, and clinical applications. J. Child Psychol. Psychiatry 42, 3–48. 10.1017/s0021963001006552 11205623

[B123] Tronick E. (2007). The Neurobehavioral and Social-Emotional Development of Infants and Children. New York, NY: W.W. Norton & Company.

[B124] Tzschoppe J. Nees F. Banaschewski T. Barker G. J. Büchel C. Conrod P. J.. (2014). Aversive learning in adolescents: modulation by amygdala-prefrontal and amygdala-hippocampal connectivity and neuroticism. Neuropsychopharmacology 39:875. 10.1038/npp.2013.287 24126454 PMC3924522

[B125] Van Overwalle F. (2009). Social cognition and the brain: a meta-analysis. Hum. Brain Mapp. 30, 829–858. 10.1002/hbm.20547 18381770 PMC6870808

[B126] Visintin E. De Panfilis C. Amore M. Balestrieri M. Wolf R. C. Sambataro F. (2016). Mapping the brain correlates of borderline personality disorder: a functional neuroimaging meta-analysis of resting state studies. J. Affect. Disord. 204, 262–269. 10.1016/j.jad.2016.07.025 27552444

[B127] Wei L. Duan X. Yang Y. Liao W. Gao Q. Ding J. R.. (2011). The synchronization of spontaneous BOLD activity predicts extraversion and neuroticism. Brain Res. 1419, 68–75. 10.1016/j.brainres.2011.08.060 21937025

[B128] Wei S. Su Q. Jiang M. Liu F. Yao D. Dai Y.. (2016). Abnormal default-mode network homogeneity and its correlations with personality in drug-naive somatization disorder at rest. J. Affect. Disord. 193, 81–88. 10.1016/j.jad.2015.12.052 26771948

[B129] Whitfield-Gabrieli S. Moran J. M. Nieto-Castañón A. Triantafyllou C. Saxe R. Gabrieli J. D. (2011). Associations and dissociations between default and self-reference networks in the human brain. Neuroimage 55, 225–232. 10.1016/j.neuroimage.2010.11.048 21111832

[B130] Wicker B. Ruby P. Royet J. P. Fonlupt P. (2003). A relation between rest and the self in the brain? Brain Res. Rev. 43, 224–230. 10.1016/j.brainresrev.2003.08.003 14572916

[B131] Zhu Y. (2004). Neuroimaging studies of self-reflection. Prog. Nat. Sci. 14, 296–302. 10.1080/10020070412331343511

